# Trends in drop out, drug free discharge and rates of re-presentation: a retrospective cohort study of drug treatment clients in the North West of England

**DOI:** 10.1186/1471-2458-6-205

**Published:** 2006-08-11

**Authors:** Caryl M Beynon, Mark A Bellis, Jim McVeigh

**Affiliations:** 1 Centre for Public Health, Faculty of Health and Applied Social Sciences, Liverpool John Moores University, Castle House, North Street, Liverpool, L3 2AY, UK

## Abstract

**Background:**

Governments aim to increase treatment participation by problematic drug users. In the UK this has been achieved by fiscal investment, an expanded workforce, reduced waiting times and coercive measures (usually criminal justice (CJ) led). No assessment of these measures on treatment outcomes has been made. Using established monitoring systems we assessed trends in 'dropped out' and 'discharged drug free' (DDF), since the launch of the national drug strategy, and rates of treatment re-presentation for these cohorts.

**Methods:**

A longitudinal dataset of drug users (1997 to 2004/05, n = 26,415) was used to identify people who dropped out of, and were DDF from, services for years 1998 to 2001/02, and re-presentations of these people in years to 2004/05. Trends in drop out and DDF, baseline comparisons of those DDF and those who dropped out and outcome comparisons for those referred from the CJ system versus other routes of referral were examined using chi square. Logistic regression analyses identified variables predicting drop out versus DDF and subsequent re-presentation versus no re-presentation.

**Results:**

The proportion of individuals dropping out has increased from 7.2% in 1998 to 9.6% in 2001/02 (P < 0.001). The proportion DDF has fallen from 5.8% to 3.5% (P < 0.001). Drop out was more likely in later years, by those of younger age and by CJ referrals. The proportion re-presenting to treatment in the following year increased from 27.8% in 1998 to 44.5% in 2001/02 (P < 0.001) for those DDF, and from 22.9% to 48.6% (P < 0.001) for those who dropped out. Older age and prior treatment experience predicted re-presentation. Outcome (drop out or DDF) did not predict re-presentation.

**Conclusion:**

Increasing numbers in treatment is associated with an increased proportion dropping out and an ever-smaller proportion DDF. Rates of drop out are significantly higher for those coerced into treatment via the CJ system. Rates of re-presentation are similar for those dropping out and those DDF. Encouragingly, those who need to re-engage with treatment, particularly those who drop out, are doing so more quickly. The impact of coercion on treatment outcomes and the appropriateness of aftercare provision require further consideration.

## Background

In many countries the health, social and criminal justice consequences of problematic drug use create an economic burden estimated at between 0.5% and 1.3% of gross domestic product [1]. Providing drug treatment programmes for drug users is considered both a cost effective and humanitarian response [2]. In the UK, treatment is seen as the cornerstone for tackling problematic drug use and increasing the number of drug users in structured treatment continues to be a key aim of the national drug strategy [3].

Internationally, governments employ diverse methods to attract drug users into treatment. Across a range of developing countries (as an example Thailand [4]), and increasingly throughout the developed world, recruitment into treatment is being achieved through compulsory or quasi-compulsory drug treatment orders issued through the criminal justice system [5]. In the United States, for example, drug courts are solely used to divert drug-using offenders away from prison into programmes involving drug testing, treatment, supervision and court-mandated sanctions for non-compliance. The number of drug courts has increased dramatically in the US since the mid 1990s [6], with other countries subsequently adopting similar models [7,8].

In the UK, a number of programmes have been initiated since the launch of the national drug strategy in 1998 to increase the number of drug users accessing treatment. Initially, massive additional funding expanded treatment provision and consequently reduced treatment waiting times. Secondly, in recognition of increasing crack use within the UK, the link between crack use and (violent) crime and the reliance in the UK on traditional substitute opiate prescribing services, specific treatment services for stimulant users were established [9]. These measures have been supplemented by a number of systems to encourage drug-using offenders to participate in treatment. Drug Treatment and Testing Orders (DTTOs), criminal justice led abstinence-focused interventions managed by the Probation Service, were introduced in 2000. The same year saw the launch of England's Arrest Referral initiative, a scheme which placed drugs workers in custody suites to ask arrestees about their drug use and to refer those who admitted use to treatment providers [10]. Although participants in Arrest Referral might receive lesser sentences for entering treatment, more recent interventions have even greater coercive elements with lesser judicial penalties in return for treatment compliance [11].

In 2003 the plethora of criminal justice based initiatives were integrated into one overarching strategy, the Drug Interventions Programme (DIP), which annually receives approximately £165 million of funding [12]. In combination, these initiatives, both criminal justice orientated and otherwise, have successfully increased the number of people accessing treatment services [13]. However, the effects of rises in recruitment of users into drug services (coercive and otherwise) on outcomes, particularly on retention in treatment, is of increasing importance but remains poorly understood.

Cheshire and Merseyside (population 2,345,077; 4.7% of England) is a mixed rural and urban area in the North West of England with prevalence of problematic drug use (usually opiate and crack addiction) as high as 52 per 1,000 male population aged 15 to 44 years in some areas [14]. In 2004/05, 8% of all clients of structured drug treatment services within England were resident within the Cheshire and Merseyside area [15]. It is the only area of the UK that has consistently collected treatment outcome data on all clients of drug services annually since 1996 (data for 1996 are not typically reported as the system was being developed in situ this year). Therefore, it provides a unique opportunity to evaluate changes in both treatment outcomes for those in structured services and trends in overall retention during a period of major changes in drug services.

Here we consider two outcomes; 'discharged drug free', (defined as a planned discharge from treatment following cessation of drug use and treatment completion), and 'drop out', (defined as unplanned discharge from services before treatment completion). Using data from the monitoring system we test two hypotheses: firstly that measures designed to increase numbers in treatment have led to an increase in proportions dropping out of treatment and secondly, that the proportion of people discharged drug free has shown a converse trend during the same time period. We compare the characteristics of those dropping out of treatment and those discharged drug free and consider what factors predict their re-presentation at treatment in the subsequent year. Further, we test the hypothesis that the rate of re-presentation to treatment is greater for those who drop out of treatment than for those discharged drug free. Finally, for a limited group of people, we explore the relationship between referral source, criminal justice or otherwise, and treatment outcome (drop out or drug free discharge). Here we hypothesise that drop out from treatment is more common amongst those referred into treatment via the criminal justice system than through other referral sources, due to the coercive nature of criminal justice initiatives, and that drug free discharge is more common for those referred from non-criminal justice sources.

## Methods

In 1996, on behalf of the local health services, the Centre for Public Health established a monitoring system to record details of all clients at structured drug treatment services in Cheshire and Merseyside. In addition to monitoring prevalence, agency staff were asked to provide outcome data, on a six-monthly basis (1^st ^January to 30^th ^June; 1^st ^July to 31^st ^December), for each client they treated (outcomes included; 'in contact with treatment', 'lost to prison', 'discharged drug free', 'referred to another agency', 'known dead', 'dropped out of treatment' and 'not in contact with treatment, other reason' [16]). At this time the national system for monitoring drug treatment contacts was not collecting prevalence and outcome data. However, a new National Drug Treatment Monitoring System (NDTMS), based largely on the Cheshire and Merseyside system was introduced in 2001 [14] and this began collecting prevalence and outcome data on an annual cycle (1^st ^April to 31^st ^March). Thus from April 2001 the system for Cheshire and Merseyside was integrated into the NDTMS and consequently its reporting period changed. Both systems collected data on those individuals in 'structured treatment services', primarily consisting of substitute prescribing, structured counselling and abstinence based services but excluding low threshold services such as needle exchange and drop in services (for a full explanation of the treatment tiers see Models of Care for Substance Misusers [17]).

Both the Cheshire and Merseyside system and the NDTMS collect data in a pseudo-anonymous form, with each individual being identified by their attributor code, comprised of their initials, date of birth and sex. The use of this attributor code for data matching and duplicate removal was validated and can be used to anonymously track individuals across years. Both systems recorded outcome data for each individual at every agency. Whilst the NDTMS records more detailed outcome data than the system it replaced, definitions for 'drug free discharge' and 'drop out' are consistent across the systems. Due to the similarities between the two systems, data have been amalgamated into a single longitudinal dataset comprising of 26,415 individuals contacting treatment services between 1^st ^January 1997 and 31^st ^March 2005 (aged 11 to 74).

This longitudinal dataset was used to retrospectively identify end of year treatment outcomes for each individual, in annual cohorts for years 1998 to 2001/02 (1998 represented the launch of the UK Drug Strategy, and 2001/02 was chosen as a cut off to enable three years of re-presentation data to accrue). Individuals reported as having end of year outcomes of drug free discharge and drop out were identified for the years 1998 to 2001/02. The data set was used to extract their attributor code, age (at the end of each reporting period), gender and information on prior treatment naivety/experience (defined here as no treatment contact from 1997/at least one prior treatment contact from 1997 to each year they appear in the dataset). Within year baseline comparisons between all treatment clients that were discharged drug free and those that dropped out of treatment were made by gender, age group and prior treatment naivety/experience using chi squared analyses. Backwards, stepwise binary logistic regression was used to identify variables which predicted drop out (compared to drug free discharge) and variables which predicted re-presentation (compared to no re-presentation) at structured treatment the year following drop out or drug free discharge irrespective of referral source. The Hosmer and Lemeshow test was used to assess the goodness of fit of the regression models [18]. Significance was set at P < 0.05 for logistic regression analyses. The longitudinal dataset was used to calculate rates of re-presentation in the year following drop out and drug free discharge up to the year 2004/05. Chi squared for trend was used for three analyses: firstly to assess changes in the proportion of individuals dropping out of treatment each year for the years 1998 to 2001/02; secondly to assess changes in the proportion of individuals discharged drug free each year during the same period of time; and finally, to assess trends in the proportion of people re-presenting at treatment in the following year (following drug free discharge and drop out). Logistic regression analyses were undertaken using SPSS v12 software [19]. All chi squared tests were performed using EpiInfo v6 [20].

Finally, this longitudinal dataset was used to retrospectively identify episodic treatment outcomes for those referred via the criminal justice system (mainly the Probation Service, Prison Officers, the Police, the Bail Support Scheme, Arrest Referral, Youth Offending Teams, Drug Treatment and Testing Orders and the court system) and those referred from other sources (mainly self referrals, or referrals through a General Practitioner or a drug service), in annual cohorts for 1998 to 2001/02. Outcomes for all treatment episodes and new treatment episodes (defined here as treatment episodes of people not recorded in treatment the previous year) were analysed separately. Chi squared analysis was used to assess differences in drop out and drug free discharge between those referred by the criminal justice system and those referred via other sources using EpiInfo v6 [20].

Ethical approval was not sought for this study which relied on the use of routinely collected, pseudo-anonymous monitoring data.

## Results

The total number of individuals (aged 11 to 74 years) in contact with treatment services in Cheshire and Merseyside was 7594, 7261, 8166 and 8061 for the years 1998 to 2001/02 respectively. Across all years, an end of year treatment outcome was available for 71% of records. The proportion of individuals who dropped out of treatment significantly increased from 7.2% in 1998, through 6.8% in 1999 and 8.3% in 2000/01 to 9.6% in 2001/02 (*X*^2 ^trend = 22.30, P < 0.001). Conversely, the proportion of people discharged drug free significantly decreased as follows: 1998, 5.8%; 1999, 9.9%; 2000/01, 4.4%; 2001/02, 3.5% (*X*^2 ^trend = 128.45, P < 0.001).

Within year comparisons showed that gender, age group and prior treatment contact was not significantly related to whether individuals were discharged drug free or dropped out (Table 1). Across years, logistic regression analysis showed drop out was predicted by younger age and year of treatment contact. Overall people were more likely to drop out in later years (after 1998; Table 2) with the exception of 1999 when the numbers recorded in treatment actually fell slightly.

**Figure 1:**
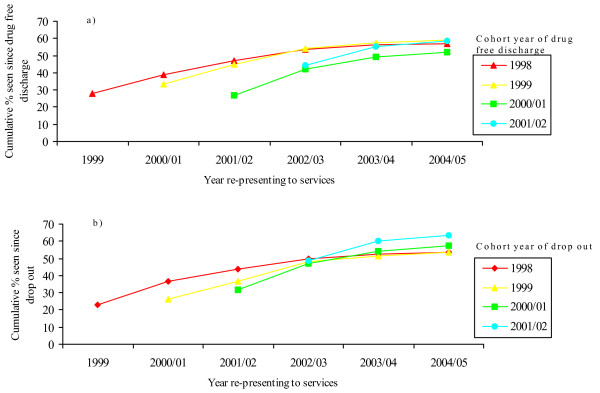
**Cumulative re-presentation rates following drug free discharge (a) and drop out (b) from structured drug treatment service**. Individuals presenting in each year of monitoring form each of the cohorts. Those discharged drug free (a) or dropping out (b) contribute to the cumulative re-presentation percentage in the first year they are seen again after leaving services. If they then leave services again they become part of a second cohort of discharge or drop out.

The proportion of those discharged drug free who then re-presented to treatment services in the following year significantly increased as follows; 1998, 27.8%; 1999, 33.6%; 2000/01, 26.9%, 2001/02, 44.5% (*X*^2 ^trend = 11.78, P < 0.001). Similarly, the proportion of those who dropped out of treatment and then re-presented in the subsequent year increased as follows; 1998, 22.9%; 1999, 26.3%; 2000/01, 31.8%; 2001/02, 48.6% (*X*^2 ^trend = 103.31, P < 0.001). Figure 1 shows cumulative rates of re-presentation to treatment services following drug free discharge and drop out for each year cohort from 1998 to 2001/02. Thus, of those discharged drug free in 1998, 57.1% had re-presented at treatment services in at least one subsequent year by 2004/05, and of those who dropped out in 1998, the equivalent proportion re-presenting by 2004/05 was 53.8%

Re-presentation to treatment in the year following treatment disengagement (of those discharged drug free and those who dropped out of treatment) was predicted by older age, having had previous treatment contact (i.e. those that were new to treatment when they dropped out were less likely to re-present) and year of treatment contact; people were more likely to re-present if discharged drug free or dropped out of treatment in 2000/01 or later than in previous years (Table 3). There was no significant difference in the rate of re-presentation, the year following treatment disengagement, for those discharged drug free compared to those who dropped out of treatment.

From 1999 and onwards, those referred into treatment via the criminal justice system were significantly more likely to drop out of treatment (and conversely significantly less likely to be discharged drug free) than those who were referred through other routes (Table 4). This difference was evident for both all treatment episodes, and treatment episodes of people who had not been recorded in treatment the previous year.

## Discussion

Investing in treatment provision is sound public health policy, with psychosocial interventions and pharmacotherapy a cost-effective alternative to non-treatment or imprisonment [21]. In the UK, treatment contact positively impacts on both health and criminal activity [22] being associated with lower levels of drug use [23], reductions in injecting, improvements in psychological health, a lower risk of non-fatal overdose [24] and lower levels of drug related crime [25]. Increasing the proportion of individuals in treatment is therefore considered to be an essential measure to tackle problematic drug use. In this respect, measures initiated in the UK to increase the capacity and availability of drug treatment in England, have been very effective, with the number of people treated in Cheshire and Merseyside rising 52% from 7594 in 1998 to 11,530 in 2004/05 (data not shown). Changes occurring in this region have been mirrored in England as a whole with numbers in treatment increasing 89% during the same period [13]. This has been achieved in part by increasing the drug treatment workforce, and consequently treatment places, a policy promoted by the National Treatment Agency for Substance Misuse following its inception in 2001. Thus, 6794 drug treatment workers were in post nationally in March 2002 compared to 10,106 in September 2005 [26]. However, the quantity of individuals in treatment is only one measure of success and needs to be contextualised with intelligence relating to treatment retention and the outcomes of individuals leaving treatment services. Recent national figures suggest that many drug users drop out long before treatment is complete, with 34% dropping out before significant benefits can accrue (suggested to be 12 weeks) [27].

Prospective cohort studies to examine outcomes are costly, time consuming and are plagued with incomplete follow up. The UK National Treatment Outcome Study (NTORS) for example, was able to follow-up 72% of its initial cohort after one year [23] and follow-up becomes increasingly problematic over longer periods of time. Use of routinely collected monitoring data from well-established systems provides an alternative means by which longer term outcomes may be assessed. It is possible, in studies of this kind that variability across years is due to the longitudinal nature of the study. However, whilst treatment outcomes have been added/removed over the years, definitions for 'drug free discharge' and 'drop out' have remained stable. Using such systems, our results suggest that increases in the numbers entering treatment are at least temporally related to increased proportions dropping out of services (7.2% in 1998 compared to 9.6% in 2001/02, P < 0.001). Conversely, there has been a trend for a decreasing proportion of people discharged drug free within the same time frame. In Cheshire and Merseyside, 5.8% of treatment clients had an end of year outcome of discharged drug free in 1998 compared to 3.5% in 2001/02 (P < 0.001). On closer examination, these increasing (drop out) and decreasing (drug free discharge) trends are only evident from 1999 onwards. 1999 saw a reduction in the number of drug users in treatment, a corresponding rise in the proportion who were discharged drug free and a fall in the proportion who dropped out. Additionally, as criminal justice based schemes were initiated in 2000, the role of the criminal justice system on treatment outcomes cannot be ignored. In 2000 onwards, the proportion of people discharged drug free at the end of the year decreased, whilst the proportion dropping out showed the opposite trend. Additionally our results show that, in 1999 onwards, those referred into treatment via a criminal justice source were significantly more likely to drop out of treatment and significantly less likely to be discharged drug free than their counterparts referred from other sources (see Table 4).

By definition, coercive strategies, criminal justice based or otherwise, force people into treatment when they are not ready to contemplate changing their drug using behaviour. Theoretically, those coerced into treatment during the 'pre-contemplation' stage of the Transtheoretical Model of behaviour change are intuitively expected to fair less well in treatment than those at the 'preparation' or 'action' stage who voluntarily self refer [28]. Additionally, whilst external motivation (e.g. the promise of bail rather than a custodial sentence) seems to promote short-term abstinence from alcohol and other drugs, internal motivation appears to be better for longer-term success. Once in treatment, practitioners therefore face the challenge of shifting a drug user's motivation from external to internal incentives [29]. If measures to increase uptake become more coercive [11] treatment must be flexible to adapt to drug users who may be very different from ones who voluntarily seek assistance. In-patient care, for example, does not have to be solely for the purpose of detoxification and drop out may be reduced if short in-patient stays were used at the beginning of treatment to identify the optimum dose of prescribed drugs [30]. Few in-patient services offer interventions for stimulant users but this group are often disproportionately represented in those recruited though criminal justice mechanisms, and may benefit considerably from such interventions [30].

It is important to note the possibility that a person's preferred main drug may be confounding the observed relationship between referral source and treatment outcome if criminal justice referrals disproportionately consist of stimulant users (particularly crack users). No proven effective pharmacological substitute exists for stimulant users as methadone exists for opiate addiction and stimulant services are in relative infancy. Less satisfactory treatment outcomes for UK methadone patients who are concurrent stimulant users has been previously suggested [31]. Two other factors are worthy of note. Firstly, population studies show that cocaine and crack cocaine use has risen in the UK since 1998 [32] and may have impacted on treatment outcomes. Secondly, opioid treatment is generally longer than treatment for stimulant addiction so, as drop out is more common than drug free discharge, it could be argued that stimulant users (referred preferentially through the criminal justice system) have greater opportunity to drop out than their non-stimulant using counterparts. However here we consider end of year treatment outcomes relating to the treatment system rather than individual treatment episodes (unless stated) with opioid and stimulant users having equal opportunity to be in contact with the system at each years' end.

Rates of re-presentation to drug services for those who dropped out of treatment and those discharged drug free have significantly increased between 1998 and 2001/02. For example, across Cheshire and Merseyside, the proportion re-presenting to treatment the year following drug free discharge increased from 27.8% for those discharged in 1998, compared to 44.5% for those discharged in 2001/02 (P < 0.001). Similarly, rates of re-presentation in the year following drop out increased from 22.9% for those dropping out in 1998 to 48.6% for those dropping out in 2001/02 (P < 0.001) (see Figure 1 for cumulative rates of re-presentation). These increases in the rate at which those leaving service re-present in subsequent years is a likely result of a combination of factors already discussed. Importantly, investing in treatment provision has considerably reduced treatment waiting times. Average waiting times have, for example halved between December 2001 and October 2004 in all treatment modalities except services focusing on abstinence or stabilisation for those with a high level of need (primarily detoxification services; tier 4 provision) [30]. People voluntarily seeking treatment have, therefore, found it easier to access services when motivation is high rather than wait until a place becomes available. Similarly, places have been available for those entering treatment via the criminal justice system. However, we also show that completion of treatment does not protect people from future drug use with rates of re-presentation being similar for those that drop out of treatment and those discharged drug free. This raises issues about the appropriateness of aftercare provision. Such services should be examined to ensure that they are seen as an integral part of the whole integrated care pathway [17], and what further measures can be taken to engage individuals who have achieved abstinence in such services. Whilst rates of relapse have been shown to be delayed for those attending aftercare services [33], treatment modalities which encourage drug free discharge, primarily in-patient detoxification facilities, must be better integrated with such services and interventions including community housing, training and support on offer [30].

Encouraging young people to engage with treatment services is a well-recognised problem [14] but reducing the time between drug initiation and treatment engagement has been recognised as important, particularly for users of more problematic drugs. Whilst a progression from abuse to dependence generally occurs over time in users of alcohol and cannabis, for cocaine and opiate users, abuse and dependence disorders tend to occur in the same year so timely intervention is essential before dependence develops [34]. Here we show that younger age predicted drop out from treatment and those in the older age groups were more likely than their younger counterparts to re-present at treatment. Current measures targeting young people therefore need particular consideration. Services established for traditional dependent opiate users may be unsuitable for some young people who are frequently users of stimulants and who perceive themselves to be drug users rather than drug dependents [34].

Drop out and relapse may be an inevitability among some client groups and in particular those who have been coerced into treatment. However if treatment is viewed from a chronic care perspective, with drug users often requiring many cycles of treatment [35], timely re-engagement becomes important to ensure that gains from previous periods of treatment are not lost. To some extent this seems to have progressed with increasing rates of re-presentation for those discharged drug free and those dropping out being observed. Re-engagement may be further improved by establishing strong linkages between structured treatment and low threshold services such as syringe exchange schemes and through effective information exchange between agencies as drug users leave one service and reappear shortly afterwards in another.

## Conclusion

In England, since 1998, huge fiscal investment in structured drug treatment provision has expanded the workforce, reduced waiting times and consequently swelled the number of problematic drug users accessing services. Such strategies have speeded up the rate at which those leaving services (following drug free discharge or drop out) re-present for a further period of treatment. Whilst drop out has always been an inevitability among this client group, we show that, in Cheshire and Merseyside, the proportion dropping out of treatment annually has also significantly increased, highlighting difficulties in retaining people in treatment. Consequently a smaller proportion of drug users are being discharged drug free. In particular, those referred via the criminal justice system are significantly more likely to drop out of treatment than those referred through non-criminal justice routes. Additionally, we show that treatment completion does not protect against further drug use, with rates of re-presentation for those discharged drug free being similar to those who dropped out of treatment suggesting that aftercare services need further consideration. Better outcomes and decreased substance use is associated with longer periods of treatment [36,37] and, if drug use is viewed as a chronic condition, treatment retention is arguably more important than drug free discharge, especially if discharge only results in relapse and further periods of chaotic drug use with associated risks. We also show that, for those leaving services, those with prior treatment history were more than twice as likely to re-present for further treatment the following year than those who were treatment naïve, suggesting that many are not overly disenchanted with the treatment they received or unwilling to try again. Measures to facilitate timely re-entry into the treatment system are important.

We conclude that recent measures to increase drug treatment participation have speeded up a revolving door both into and out of treatment. The effectiveness of aftercare services for those leaving treatment drug free and the impact of coercive measures to facilitate treatment engagement on longer-term outcomes, in particular retention, need further consideration.

## Competing interests

The author(s) declare that they have no competing interests.

## Authors' contributions

CB was involved with study design, conducted the data manipulation and statistical analysis and participated in writing the manuscript. MB assisted with study design and statistical analysis and participated in writing the manuscript. JM assisted in designing the study and in writing the manuscript. All authors read and approved the final manuscript.

## Pre-publication history

The pre-publication history for this paper can be accessed here:



## Figures and Tables

**Table 1:** Comparisons between those discharged drug free and who dropped out at each year's end by age group, sex and previous treatment contact

	Year of treatment outcome							
	
	1998		1999		2000/01*		2001/02	
	
	Discharged drug free	Dropped out	Discharged drug free	Dropped out	Discharged drug free	Dropped out	Discharged drug free	Dropped out
n (%)	443 (44.6%)	550 (55.4%)	718 (59.4%)	491 (40.6%)	360 (34.7%)	676 (65.3%)	281 (26.7%)	772 (73.3%)
< 25 years old	118 (26.6%)	166 (30.2%)	144 (20.1%)	110 (22.4%)	72 (20.0%)	114 (16.9%)	41 (14.6%)	146 (18.9%)
25 years old and over	325 (73.4%)	384 (69.8%)	574 (79.9%)	381 (77.6%)	288 (80.0%)	562 (83.1%)	240 (85.4%)	626 (81.1%)
within year comparison	P = 0.219		P = 0.325		P = 0.210		P = 0.105	
Female	130 (29.3%)	153 (27.8%)	227 (31.6%)	148 (30.1%)	109 (30.3%)	232 (34.3%)	92 (32.7%)	226 (29.3%)
Male	313 (70.7%)	397 (72.2%)	491 (68.4%)	343 (69.9%)	251 (69.7%)	444 (65.7%)	189 (67.3%)	546 (70.7%)
within year comparison	P = 0.596		P = 0.587		P = 0.187		P = 0.279	
New to treatment that year	239 (54.0%)	328 (59.6%)	324 (45.1%)	195 (39.7%)	193 (53.6%)	377 (55.8%)	118 (42.0%)	366 (47.4%)
Previously seen**	204 (46.0%)	222 (40.4%)	394 (54.9%)	296 (60.3%)	167 (46.4%)	299 (44.2%)	163 (58.0%)	406 (52.6%)
within year comparison	P = 0.072		P = 0.062		P = 0.506		P = 0.119	

*Reporting changed from UK calendar to financial year to adhere to new national requirements.

**Previously seen is defined as having had at least one prior treatment contact between 1997 and the year analysed.

**Table 2:** Factors predicting drop out from drug treatment compared to drug free discharge

Factors predicting drop out from treatment (N = 4291)		
Variable	Adjusted odds ratio (95% confidence interval)	P value

Age group		0.013
10 to 19		Ref
20 to 29	0.62 (0.45, 0.86)	0.003
30 to 39	0.68 (0.49, 0.93)	0.016
40 to 49	0.55 (0.37, 0.81)	0.003
50 to 59	0.43 (0.24, 0.76)	0.004
60 to 69	0.23 (0.02, 2.74)	0.245
Year		< 0.001
1998		Ref
1999	0.55 (0.46, 0.66)	< 0.001
2000/01*	1.53 (1.28, 1.83)	< 0.001
2001/02	2.22 (1.84, 2.67)	< 0.001

*Reporting changed from UK calendar to financial year to adhere to new national requirements.

Ref is the reference category.

Other variables entered into the analysis which were not significant were sex (P = 0.93) and new to treatment/previous treatment contact (P = 0.55).

Hosmer and Lemeshow Goodness-of-Fit Test: *X*^2 ^= 8.102, P = 0.231.

**Table 3:** Factors predicting re-presentation to treatment for those who dropped out and were discharged drug free in the previous year

Factors predicting re-presentation as treatment (N = 4291)		
Variable	Adjusted odds ratio (95% confidence interval)	P value

Age group		0.031
10 to 19		Ref
20 to 29	1.87 (1.27, 2.75)	0.002
30 to 39	1.73 (1.17, 2.55)	0.006
40 to 49	1.50 (0.95, 2.38)	0.081
50 to 59	1.38 (0.71, 2.71)	0.345
60 to 69	1.72 (0.15, 20.36)	0.667
Year		< 0.001
1998		Ref
1999	1.17 (0.96, 1.42)	0.117
2000/01*	1.28 (1.04, 1.56)	0.017
2001/02	2.60 (2.14, 3.15)	< 0.001
Treatment history		
New to treatment		Ref
Prior treatment contact	2.50 (2.18, 2.86)	< 0.001

*Reporting changed from UK calendar to financial year to adhere to new national requirements.

Ref is the reference category.

Other variables entered into the analysis which were not significant were sex (P = 0.395) and outcome (drop out versus discharged drug free) (P = 0.522).

Hosmer and Lemeshow Goodness-of-Fit Test: *X*^2 ^= 7.273, P = 0.508.

**Table 4:** Episodic drug treatment outcome (discharged drug free and drop out) by referral source

Year of treatment outcome	Referral source	Outcome of treatment episode							
		
		All episodes				Episodes for people not in treatment the previous year			
		
		Discharged drug free		Dropped out		Discharged drug free		Dropped out	
		N	%	N	%	N	%	N	%

1998	Criminal Justice	11	30.6	25	69.4	--	--	--	--
	Non-Criminal Justice	292	43.5	380	56.5	--	--	--	--
	Within year comparison	P = 0.128				--			
1999	Criminal Justice	33	37.5	55	62.5	16	27.6	42	72.4
	Non-Criminal Justice	444	59.6	301	40.4	172	66.7	86	33.3
	Within year comparison	P < 0.001				P < 0.001			
2000/01*	Criminal Justice	20	15.5	109	84.5	11	12.9	74	87.1
	Non-Criminal Justice	272	41.9	377	58.1	142	40.5	209	59.5
	Within year comparison	P < 0.001				P < 0.001			
2001/02	Criminal Justice	25	14.0	153	86.0	18	15.1	101	84.9
	Non-Criminal Justice	335	30.9	749	69.1	167	28.7	414	71.3
	Within year comparison	P < 0.001				P = 0.002			

*Reporting changed from UK calendar to financial year to adhere to new national requirements.

1998 was the first year of analysis. It is therefore not possible to differentiate between 'all episodes' and 'episodes for people not in treatment the previous year' for this annual cohort.
